# Abstract of the Proceedings of the Buffalo Medical Association

**Published:** 1856-02

**Authors:** Sanford B. Hunt


ART. II.—*Abstract of the Proceedings of the Buffalo Medical Association.*

The first Tuesday in January falling upon New Year's Day, the association met at its rooms on the evening of Wednesday, Jan. 2d, 1856.

Present—The President, Dr. Strong, Wyckoff, Hunt, Wilcox, Underhill, Miner, Newman, Hutchins, Nott, White, Rochester, Ring, Eastman, Lockwood, Gay, Lay, and Gray.

*Dr. Rochester* presented a pathological specimen. The patient from whom it was obtained, Dennis Murphy, a sailor, aged 45 years, of dissipated habits, was admitted to the general ward of the hospital, in November. He had œdema of the extremities, which did not seem to depend upon any disease of the heart or kidneys, both of these organs proving healthy upon examination. He suffered from nausea and vomiting, with dull pain both in the head and in the region of the liver. On examination the region of hepatic dullness was found diminished. The chest, upon percussion, was clear as low as the eighth rib, and the hand could be carried beneath the free margin of the ribs for some distance without feeling the liver. There was no evidence of disease in the chest, save a very general bronchitis of a chronic form. After his admission the symptoms increased, and he coughed or vomited much blood, which may have come from either the stomach or lungs. It resembled the blood of hæmatemesis, and he had anorexia, nausea and tenderness in the epigastrium. There was, however, a severe cough, with dyspnœa. The diagnosis of the case was cirrhosis.

On examining the sputa one morning, Dr. R. found, among some blood and mucc-purulent matter, the specimen exhibited, which seemed to be a fibrinous cast of the right bronchus with two of its branches. It consisted of very firm false membrane, was about two inches in length, and of sufficient width to cover the whole circumference of the bronchus. He could not be positive as to the source of this membrane, but the dyspnœa was much, and the cough somewhat, relieved after its expulsion. The patient had recently left the hospital and resumed his intemperate habits, so that Dr. R. was unable to speak of his present condition. There is a similar specimen at the Medical College, in Dr. Flint's collection, which was coughed up by a tubercular patient at Lockport.

*Dr. Hunt* reported a case of tænia lata. —— Godfrey, aged 26 years, a native of county Antrim, Ireland, a saddler by trade, was admitted to the hospital, Dec. 24th. Dr. Rochester being absent for the holidays, the patient fell under the charge of Dr. Hunt. The history was as follows: Three years ago, when in Ireland, he had passed joints of the tape-worm. Many others in his neighborhood had tape-worm about that time. He applied to a physician who gave him a purgative mixture containing oil of turpentine, which brought away a considerable length of the worm. Since that time, however, he had occasionally passed joints of the worm, and had suffered much from general malaise, variable and capricious appetite, occasional diarrhœa, nausea, vertigo and headache. The pupils of the eyes were unnaturally dilated, and he complained also of dragging pains in the loins, and cold feet. He had a peculiar sensation in the region of the descending colon which, whether imaginary or not, seemed to be the motion of the parasite.

The following prescription was ordered Dec. 25: To eat no supper that night, and, fasting in the morning, to take the following orgeat:

Take of the seeds of the pumpkin, ℥ij. Bruise thoroughly in a mortar, add enough boiling water to make 8 ounces, and take at once.

This was followed by the administration of castor oil, ℥ij., two hours after the above.

On the operation of the oil a specimen of tænia lata was brought away about five feet in length. There was some question as to whether the head was obtained. On examination with a lens, the neck was found terminating in a small conical point, not abrupt and broken like the joints, but smooth and rounded. As the specimen had been unfortunately frozen, it was impossible to decide positively that this was the head.

On the 26th, Dr. Rochester returning, resumed charge of the ward, and continued the treatment, but without further result.

*Dr. Strong* inquired whether any confidence was to be placed in the newspaper opinion that cases of tape-worm are confined to pork eaters.

*Dr. Rochester* thought not. Tape-worm was not more prevalent in Ohio than elsewhere, while in Abyssinia, where little pork is eaten, it is a very frequent disease.

*Dr. White* also instanced cases of tape-worm in delicate females who had a strong aversion to pork.

*Dr. Nott* reported a peculiar case of erysipelas. A Welchman, aged 23, of robust habit, a farmer, had noticed that his teeth were loose (the incisors of the lower jaw) for three days previous to that on which Dr. Nott was called. During this time he had continued to labor. Dr. N. found that he had had diarrhœa the day before, that the teeth of the lower jaw, on the right side, were loose, with a sanious pus issuing from the gums. There was not much swelling of the throat internally or externally. On the second day he was delirious, the swelling had extended to the upper jaw and roof of the mouth, and the sanious discharge continued. On the third day of the attendance, Dr. Rochester was called in consultation. He was at this time comatose, had tenderness of the epigastrium, vomiting, and typhoid symptoms generally. His articulation was indistinct, and a fœtid sanies discharged from his mouth. From this time he continued to improve for a few days. Dr. Nott saw him Nov. 6th, at 2, P. M. He was then sitting up, his teeth were firmer in their sockets, but he had still some difficulty in swallowing. At 9, P. M., of the same day, a noise was heard in his room, and on entering it he was found dead. There was some œdema of the face.*

*Dr. Gray* reported a similar case of a boy aged 8 years, who had been sick for three or four days when Dr. G. was called. Found him with a swelling of the chin and lower jaw of a shining white appearance. Next

* Query.—Œdema glottidis?—*Sec.*

morning he had excessive pain in the chin, with a very offensive sanious discharge from the gums, which were swollen and spongy, and the four incisor teeth were loose. The patient was cachectic, had been feeble for three or four weeks, the pulse was feeble and frequent, the nails were crumbly, one-half of the nail being gone on two or three of the fingers. He recovered under a tonic and anodyne treatment, with the local use of chloride of soda.

*Dr. White* reported a case of a gentleman aged fifty-five, an intelligent business man cf active habits, who in previous years had suffered much from rheumatism. He went to Wisconsin some two years since. Two months ago his friends brought him home paralytic. He had been treated for paralysis in Wisconsin; strychnia, among other remedies, had been given without avail. At the time Dr. W. was called he was to a great degree demented, unable to recognize his friends, while the paralysis was general, though affecting the right side most. He had much headache, bowels costive, urine passed unconsciously. He was at times very stupid, but when not in that condition he suffered much from hyperæsthesia of the surface. The paralysis seemed more sensitive than motor, for he was able to sit up in a chair, and, though very awkward, to grasp with considerable firmness.

A mixture of ol. tiglii, ol. terebinth, and copaiba was given as a cathartic, sinapisms were applied, and iron, quinine, and iodide of potassium given internally. After he had been a week or two under treatment, Dr. Hunt was invited to see him. Dr. H. regarded the case as hopeless without making any definite diagnosis. Notwithstanding these unfavorable opinions, the patient began to amend, and has continued steadily improving. After six or seven weeks of treatment he seems restored, his mind is cheerful, he recognizes friends readily, walks about the streets, converses well, his bowels have become regular, and he has regained his control of the bladder.

*Dr. Wilcox* related a case of a young man, a brass-founder, whose occupation was burnishing and silvering bells, who had ulceration of the nostrils with epistaxis, and had become quite exsanguine. Attributing the disease to his occupation, he was directed to discontinue work, and take quinine and iron. Last Sunday he presented himself supposing that he was salivated. His incisor teeth of the lower jaw were loose, and there was a sanious discharge from the gums. This seems to have been corrected by the local application of tr. myrrhæ. The case would not have attracted his attention particularly, were it not for the analogous cases reported by Drs. Nott and Gray.

*Dr. White* gave a brief history of the case of a young German mechanic, of active aad industrious habits. He had been attending a series of religious meetings, and was said to have had typhoid fever previously. He came under Dr. W.'s charge as a private patient at the hospital; was then emaciated, and suffering under religious monomania, refusing to speak or eat. His symptoms were analogous to those of hysteria. The patient continuing to refuse food, and to speak, Dr. W. called Dr. Rochester in consultation, and it was agreed between them, *in presence of the patient*, that the actual cautery should be applied on the succeeding day, great pains being taken to enlarge especially on the details and painful character of the operation. This *ruse* had the desired effect, but the patient then talked too much, and annoyed the house by his constant vociferation. For this he was subjected to the cold douche upon the head, continued until he promised amendment. The patient looked upon this, not as a remedial measure, but as a necessary penance for his sins. Under the use of tonics directed to the nervous system, he regained his health both of mind and body.

*Dr. Gay* mentioned an amusing case of hysterical delusion in a patient who had been previously insane. She refused nourishment, and actually passed a week without food. Dr. G. resorted to a *ruse*, and telling the husband, in the patient's presence, that she must be mesmerized, he proceeded to make the usual "passes," and then calling for food, pronounced her "mesmerized," and fed her *ad libitum.*

*Dr. Nott* reported a case of a young ship-carpenter, who applied to him in the night for relief from an excessive neuralgic pain in the right arm, which had then continued for two nights. His bowels were open, he had no spinal tenderness, and his appetite was good. Prescribed

℞ Chloroform, camphorat, ℥iv.

Ol. Sinapis Æth., M. LX.

He had no more pain after using this, but complete anæsthesia followed. For this he took blue pill for a day or two, continuing the liniment. The thumb recovered first from the insensibility, and now only two joints of the middle finger continue numb.

*Dr. Newman*, from the committee on organization, reported that Dr. Baker had it in charge, and would soon report.

Adjourned.

SANFORD B. HUNT, Secretary.

